# Down-Regulation of Hydrogen Sulfide Biosynthesis Accompanies Murine Interstitial Cells of Cajal Dysfunction in Partial Ileal Obstruction

**DOI:** 10.1371/journal.pone.0048249

**Published:** 2012-11-01

**Authors:** Xin Guo, Xu Huang, Yi-song Wu, Dong-hai Liu, Hong-li Lu, Yong-chul Kim, Wen-xie Xu

**Affiliations:** 1 Department of Physiology, Shanghai Jiaotong University School of Medicine, Shanghai, China; 2 Department of Urology, Affiliated Hospital of Nantong University, Nantong, China; 3 Department of Physiology, Chungbuk National University College of Medicine, Cheongju, Republic of Korea; Juntendo University School of Medicine, Japan

## Abstract

**Purpose:**

To investigate the role of endogenous hydrogen sulfide (H_2_S) in partial obstruction-induced dysfunction of the interstitial cells of Cajal (ICC) in mice ileum.

**Materials and Methods:**

Partial intestinal obstruction was induced surgically in male imprinting control region (ICR) mice. ICC networks were studied by Immunohistochemistry. Electrical activity was recorded by intracellular recording techniques. The expression of ICC phenotype marker c-kit receptor tyrosine kinase (c-kit), membrane binding stem cell factor (mSCF), the endogenous H_2_S biosynthesis enzymes cystathionine-β-synthase (CBS) and cystathionine-γ-lyase (CSE) was studied by Western blotting. The expression of tumor necrosis factor-α (TNF-α) mRNA was observed by using real-time polymerase chain reaction.

**Results:**

Partial intestinal obstruction resulted in ICC networks were disrupted above obstruction 14 days after the operation. The slow waves of intestinal smooth muscles in the dilated region were significantly suppressed and their amplitude and frequency were reduced, whilst the resting membrane potentials were depolarized. The expression of c-kit and mSCF was significantly decreased, also suggesting the disruption of the ICC network. The expression of TNF-α was significantly increased in the tunica muscularis of the obstructed intestine. Treatment of cultured intestinal smooth muscle cells with TNF-α caused dramatic down regulation of mSCF. The expression of CBS and CSE was significantly decreased in the tunica muscularis of the obstructed intestine. Intraperitoneal injection (i.p) of DL-propargylglycine, an irreversible inhibitor of CSE, and aminooxyacetic acid, an inhibitor of CBS, elevated the expression of TNF-α mRNA in the tunica muscularis of the ileum. Obstruction-induced over expression of TNF-α was significantly improved by supplementation of NaHS, but not the expressions of mSCF and c-kit.

**Conclusions:**

The down regulation of endogenous H_2_S biosynthesis is related to over expression of TNF-α in obstructed small intestine. TNF-α-mediated mSCF down-regulation is not the only reason of partial intestinal obstruction-induced loss of ICC.

## Introduction

Several congenital or acquired digestive tract motility disorders are associated with localized intestinal obstruction. Partial obstruction of the intestine has been shown to induce distention and hypertrophy of the gastrointestinal (GI) smooth muscles above the site of occlusion in both clinical situations and animal models [1], [2]. Partial obstruction causes dramatic changes in intestinal gross morphology, interstitial cells of Cajal (ICC), and the networks and ultrastructure of smooth muscle cells [3]–[5]. However, little is known about the molecular changes that occur in intestinal smooth muscles in response to mechanical obstruction [6], [7].

In the gastrointestinal tract, the distribution of ICCs throughout the musculature is associated with nerve structures. ICCs generate a periodic depolarization at a characteristic frequency that is called the slow wave or pacemaker activity. This involves rhythmic oscillations of intracellular calcium and activation of membrane ion channels [7]–[9]. Importantly, ICCs express c-kit receptor tyrosine kinase. In the GI tract, development and maintenance of the ICC phenotype have been linked to intracellular signaling via c-kit [9]–[11]. Previous studies have indicated that ICCs are lost in partial mechanical obstruction of the GI tract, and the severest damage to the ICC network is found in the region immediately above the obstructed site [5], [8]. Maintenance of the ICC network requires membrane-bound stem cell factor (mSCF) produced locally by the intestinal smooth muscles [12]–[15]. Previous studies have shown that the distended region of the intestine demonstrates inflammatory reactions, which could cause the disruption of the ICC network [8], [16], [17]. The proinflammatory cytokine tumor necrosis factor-α (TNF-α) has been shown to be a central mediator of inflammation in the obstructed ileum. TNF-α has led to increased inflammatory reactions that result in the development of comprehensive muscularis inflammation in the dilated region of the intestine in obstructed mice [8]. However, it is not clear how TNF-α causes the disruption of the ICC network.

Hydrogen sulfide (H_2_S) is a pungent gas that is formed endogenously in mammalian tissues by the H_2_S biosynthesis enzymes cystathionine-β-synthase (CBS) and cystathionine-γ-lyase (CSE) [18], [19]. H_2_S biosynthesis has been found in the gastrointestinal tract [20], [21]. A number of putative physiological and pathophysiological roles for this gas have been put forward, and a range of potential therapeutic uses of this gas have been proposed [22], [23]. It is recognized that H_2_S exerts complex effects on inflammation. Administration of sodium hydrosulfide (NaHS), a “fast releasing” H_2_S donor, provoked an inflammatory reaction in mice [24]. However, NaHS has been reported to inhibit leukocyte adhesion to gastric mucosal blood vessels, which suggests an anti-inflammatory effect [25]. Morpholin-4-ium 4 methoxyphenyl (morpholino) phosphinodithioate (GYY4137) has been reported to slowly release H_2_S over a period of hours both in vitro and in vivo. GYY4137 exhibits anti-inflammatory activity as evidenced by a reduction in the lipopolysaccharide (LPS)-induced increase in plasma proinflammatory cytokines (TNF-α, IL-1b, IL-6), nitrite/nitrate, C-reactive protein, and L-selectin in conscious rats [24]. Therefore, H_2_S exerts complex and at times opposing effects on inflammation in whole animals [24], [25]. These observations led us to investigate the role of endogenous H_2_S in the inflammatory cytokine TNF-α expression in the obstructed intestine.

## Materials and Methods

### Experimental Model

Male imprinting control region (ICR) mice (5-weeks-old, 30 ± 2 g) were obtained from the Experimental Animal Center of Shanghai Jiaotong University School of Medicine. This study was carried out in strict accordance with the recommendations in the Guide for the Care and Use of Laboratory Animals of the Science and Technology Commission of P.R.C. (STCC Publication No. 2, revised 1988). The protocol was approved by the Committee on the Ethics of Animal Experiments of Shanghai Jiaotong University School of Medicine (Permit Number: Hu 686-2009). All surgery was performed under ether, and all efforts were made to minimize suffering. A total of 57 mice were used in our study. Sixteen mice were used in the obstructed model research and were divided into control group and obstructed group. 16 mice were used to study the role of endogenous H_2_S in obstruction-induced intestinal inflammation and were divided into control group and treatment group. 24 mice were used to study the effect of supplementation of H_2_S on expressions of TNF-α mRNA, mSCF and c-kit proteins in obstructed intestinal smooth muscle tissues. One mouse was used for intestinal smooth muscle cell culture.

Partial intestinal obstruction was induced by surgically placing a ring of silicon tube around the ileum 30–50 mm oral to the ileocecal sphincter to cause distension of the intestine above the site of obstruction according to the methods described by Chang et al [5] and Won et al [8]. Experiments were performed with distended segments of ileum 14 days after surgery. Control and obstruction mice were anaesthetized with ether and euthanized by cervical dislocation. A 50-mm segment of small intestine oral to the obstruction was removed and pinned out in the base of a Sylgard silicone elastomer dish containing Krebs of the following composition (mM): NaCl 118.5; KCl 4.5; MgCl_2_ 1.2; NaHCO_3_ 23.8; KH_2_PO_4_ 1.2; glucose 11.0; CaCl_2_ 2.4. The segment was opened by cutting lengthwise, washed with Krebs, and the mucosa and submucosa removed by sharp dissection [5], [26]. The remaining tunica muscularis was used for electrophysiological recordings. Some of the tissues were quickly frozen in liquid nitrogen and stored at −80°C for later use.

To investigate the role of endogenous H_2_S in obstruction-induced intestinal inflammation, we used DL-propargylglycine (PAG, 50 mg/kg), an irreversible inhibitor of CSE, and aminooxyacetic acid (AOA, 17 mg/kg), an inhibitor of CBS, to inhibit H_2_S biosynthesis. The mixture solution of PAG and AOA was administered via intraperitoneal injection (i.p) twice a day for four days. The control mice were injected with same volume of phosphate-buffered saline (PBS).

To observe the effect of supplementation of H_2_S on expressions of TNF-α mRNA, mSCF and c-kit proteins in obstructed intestinal smooth muscle tissues, obstructed mice model was established. The solution of NaHS (10 umol/kg) [27] was administered via i.p once a day for nine days from the fifth day of obstruction. The control mice were injected with same volume of PBS.

### Fluorescent Immunohistochemistry

Smooth muscle strips were stretched to 110% of their resting length, fixed with ice-cold acetone (4°C; 10 min), and processed as whole mounts. Following fixation, preparations were washed for 60 min in phosphate-buffered saline (PBS; 0.01 M, pH 7.4). Tissues were then incubated in 10% goat serum containing 0.3% Triton X-100 for 1 hour at room temperature to reduce nonspecific antibody binding [5], [8]. For examination of ICCs, tissues were incubated overnight at 4°C with polyclonal (rabbit) c-Kit antibodies (sc-5535; Santa Cruz Biotechnology, Santa Cruz, CA, USA; 1∶200 dilution). Immunoreactivity was detected using fluorescent isothiocyanate (FITC-488)-conjugated secondary antibody (FITC–anti-rabbit lgG; Santa Cruz Biotechnology, Santa Cruz, CA, USA; 1∶400 dilution in PBS, 1 h, room temperature).

### Electrophysiological Experiments

Strips of smooth muscle (8 mm×4 mm) were cut parallel to the longitudinal axis of the intestine, oral to the site of occlusion. The smooth muscles were placed in a recording chamber with the submucosal aspect of the muscle facing upwards at 37°C in an atmosphere of 95% O_2_ and 5% CO_2_. Cells were impaled with KCl-filled glass microelectrodes with resistances of 50–90 MΩ. Electrical responses were recorded and amplified through a high input impedance amplifier (SYS-773 Duo 773 Electrometer, WPI, USA). Experiments were performed in the presence of nifedipine (1 µM; Sigma, St Louis, MO, USA) in the perfusion solution to reduce contraction and facilitate cell impalement. Slow waves in mouse intestine have been previously shown to be unaffected by nifedipine [28].

### Western Blotting

Tunica muscularis or cultured intestinal smooth muscle cells were lysed in RIPA buffer (25 mM Tris-HCl pH 7.6, 150 mM NaCl, 1% NP-40, 1% sodium deoxycholate, 0.1% SDS). Protein concentration was determined using the Bradford method. The lysate (20 µg of total protein per lane) was subjected to electrophoretic separation with 10% SDS–PAGE and transferred to nitrocellulose membranes (Hybond, Amersham). The membrane was incubated with 5% milk to reduce nonspecific antibody binding. Western blot was performed using antibodies directed against c-Kit (1∶200 dilution, rabbit polyclone, Santa Cruz Technologies), mSCF (1∶200 dilution, mouse monoclone, Santa Cruz Technologies), CBS (1∶200 dilution, mouse monoclone, Santa Cruz Technologies), CSE (1∶200 dilution, mouse monoclone, Santa Cruz Technologies), and GAPDH (1∶600 dilution, rabbit monoclone, CW Biotech Company, Beijing, P.R. China). Alkaline phosphatase (AP) conjugated secondary antibodies and BCIP/NBT alkaline phosphatase color development kit (CW Biotech Company Beijing, P.R. China) were used to detect protein bands. The images were captured by Adobe Photoshop and analyzed with Quantity One image software [29].

### Real-time Polymerase Chain Reaction

The mRNA expression of TNF-α in intestinal smooth muscle tissue was detected by real-time polymerase chain reaction (RT-PCR). Specific primers for mouse TNF-α were 5′ -GACGTGGAACTGGCAGAAGAG- 3′ and 5′ - TTGGTGGTTTGTGAGTGTGAG - 3′, (228 bp). Amplification was performed in parallel samples using glyceraldehyde-3-phosphate dehydrogenase (GAPDH, 496 bp) as a control. Briefly, total RNA was isolated from smooth muscle tissue as recommended by the manufacturer of TRIzol Reagent. RNA concentration was determined by reading absorbance at 260/280nm and was adjusted to 0.4 µg/µL. Reverse transcription was performed according to the manufacturer’s instructions. cDNA samples were used for PCR using specific primers for TNF-α. The following conditions were used for PCR amplification: 95°C for 4 min; 95°C for 30 sec; followed by 38 cycles at 58°C for 1 min; 72°C for 30 sec; 72°C for 7 min. The PCR products were separated on a 2% agarose gel. Possible contamination of DNA was tested by skipping the reverse transcription step prior to PCR. Detectable fluorescent bands were visualized by an ultraviolet transilluminator (Bio-Rad) and the area was measured using Quantity One image software [29].

### Isolation and Culture of Mouse Intestinal Smooth Muscle Cells

Intestinal smooth muscle cell (ISMC) culture was prepared from mouse intestine using an explant technique [29]. Briefly, the smooth muscle layer of the mouse intestine was surgically isolated and minced into small pieces. The tissue suspension was plated onto 25 cm^2^ culture flasks for culture in DMEM containing 10% heat-inactivated fetal bovine serum (FBS), 2 mmol/L glutamine, 100 mmol/L HEPES, 100 U/mL penicillin, 100 mg/mL streptomycin, and incubated at 37°C in a humidified incubator containing 5% CO_2_. ISMCs between passages 5–7 were used and displayed typical spindle shape morphology and a “hill-and-valley” pattern of growth. ISMCs were characterized by immunohistochemical staining for smooth muscle-specific α-actin (Santa Cruz Biotechnology, Santa Cruz, CA, USA). DAPI was used for nuclear staining [29].

ISMCs were seeded into 6-well plates at a density of 1×10^6^ cells/well and incubated in 1 mL of serum-supplemented DMEM (10% FBS, 1% penicillin/streptomycin) for 48 hours. Thereafter, the cell layers were washed with PBS and incubated for 24 hours in 1 mL of serum-free DMEM. After 24 hours, the cells were washed with PBS and incubated for 24 hours in 1 mL of serum-free DMEM containing 10 ng/mL of TNF-α (R&D Systems GmbH, Wiesbaden-Nordenstadt, Germany) [30]. Cells were then harvested for Western blotting.

### Statistical Analysis

Data were expressed as means ± SE. Student’s *t*-test was used to determine statistical significance. *P*<0.05 was considered statistically significant.

## Results

### Changes in Intestinal Morphology and Electrophysiological Activity

**Figure 1:**
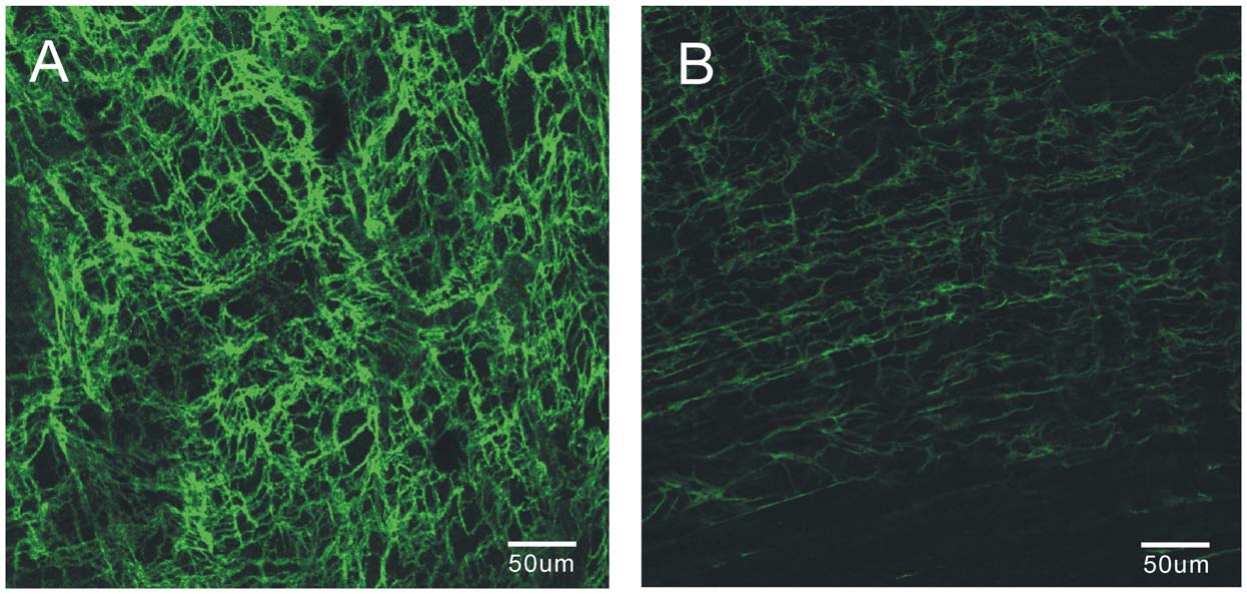
Fluorescent Immunohistochemistry of c-Kit in whole smooth muscle preparations. The control exhibited a dense network in the interstitial cells of Cajal (A), where this network was disrupted in the obstruction mice whole smooth muscle tissue (B).

**Figure 2:**
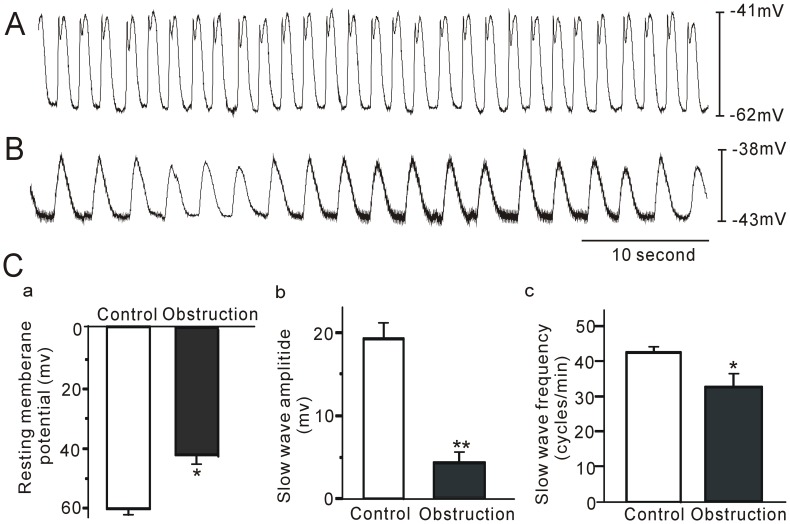
Electrical slow waves recorded from intestinal smooth muscle tissues. The control (A) and obstructed murine model (B). A summary of electrical parameters from control and the 14-day partial obstruction are shown in (C). C-(1), resting membrane potential (RMP); C-(2), slow wave amplitude; C-(3), slow wave frequency. *P<0.05; **P<0.01; n = 8.

**Figure 3:**
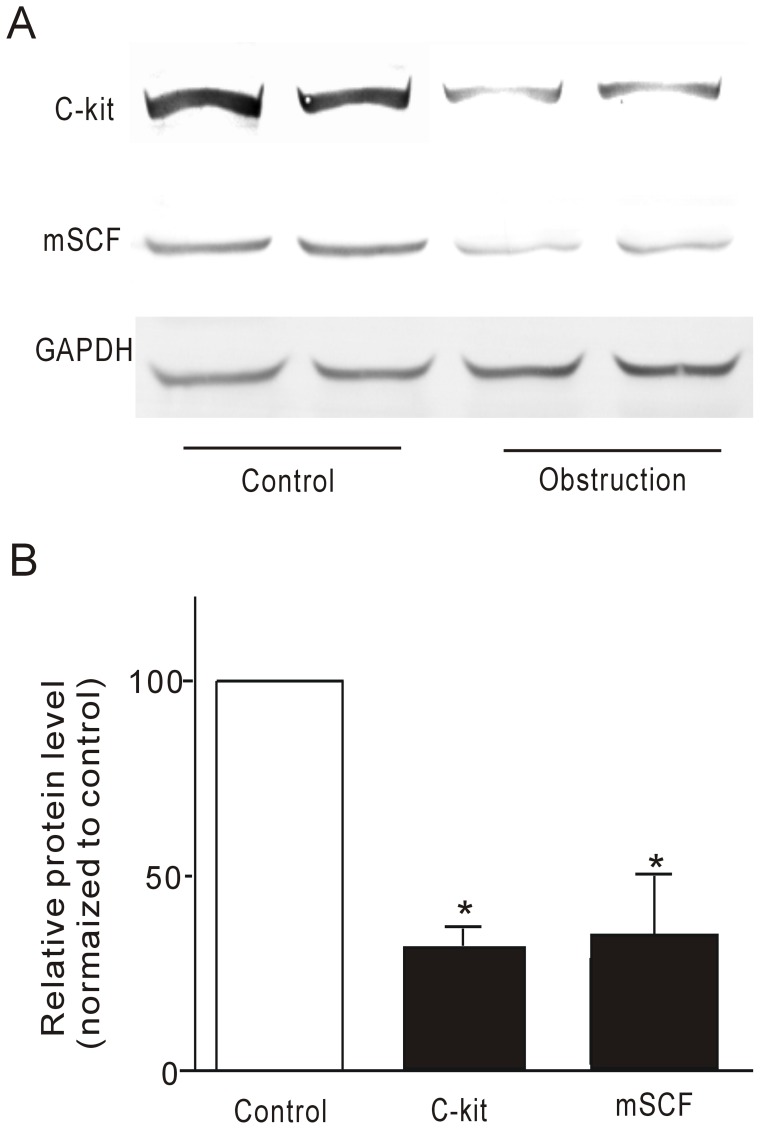
Western blot revealed different expression of mSCF and c-Kit between control and obstructed ileal smooth muscle tissues. A representative blot is shown in (A), whilst the densitometric quantification (%GAPDH and normalized to control) is depicted in (B). **P<0.01, n = 8.

Partial ileal obstruction causes intestinal distention and thickening of smooth muscle layer (Figure S1) and the disrupted of ICCs (Fig. 1B) above the site of occlusion. The changes were similar to those presented previously [5]. In the gastrointestinal tract, ICCs within the smooth muscle layer generate slow waves, which elicit and control spontaneous smooth muscle contraction. Slow waves were, therefore, recorded from circular muscle strips in control and obstructed mouse intestine (Fig. 2A, 2B). The resting membrane potential (RMP) in intestinal smooth muscle cells was −61.4±2.1 mV in the control group, and became depolarized to −42.8±2.5 mV (P<0.05) in the obstructed group (Fig. 2C-1). The amplitude of slow waves varied from 19.2±1.0 mV in controls to 4.2±1.3 mV (P<0.01) in the obstructed group (Fig. 2C-2). The frequency of slow waves was 42 ± 1.3 cycles/min in controls and declined to 32.8±1.1 (P<0.05) cycles/min in the obstructed group (Fig. 2C-3). These results suggest alterations of ICC function in the obstructed ileum.

### Effect of Partial Obstruction on the Expression of mSCF and c-Kit

Previous studies demonstrated that ICCs were lost from the intestinal region immediately oral to the partial mechanical obstruction [5]. However, maintenance of ICCs requires mSCF produced locally by the intestinal smooth muscles [31]. Therefore, the protein expression of mSCF and c-Kit was observed in partially obstructed ileum by Western blot. Representative immunoblots for mSCF, c-Kit and GAPDH from control and obstructed groups are shown in Figure 3A. Immunoblots for mSCF and c-Kit indicated the presence of mSCF and c-Kit proteins in the smooth muscle tissue preparations. The amount of mSCF and c-Kit proteins was significantly decreased in the obstructed group (Fig. 3B).

### Effect of Partial Obstruction on the Expression of TNF-α

**Figure 4:**
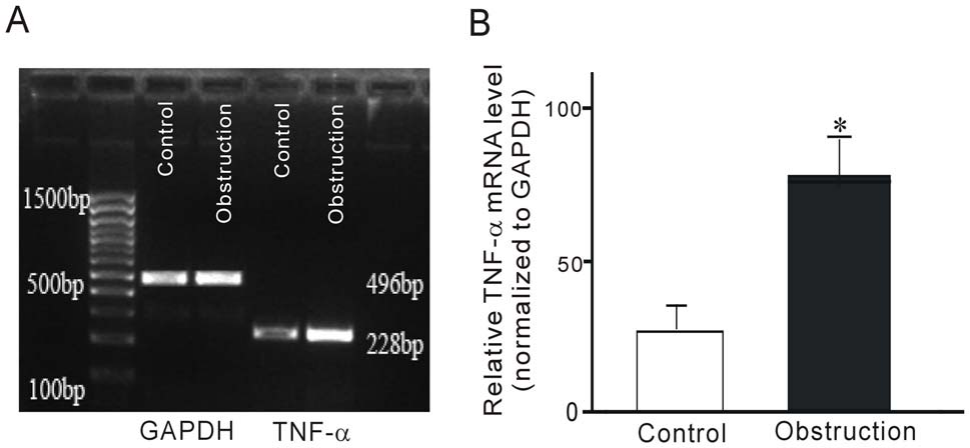
Expression of TNF-α mRNA in obstructed ileal smooth muscle tissues. (A) Expression of TNF-α and GAPDH mRNA. (B) Densitometric analysis of TNF-α and GAPDH mRNA. **P<0.01, n = 8.

Won et al [8] found that mRNA expression of TNF-α was significantly increased in the obstructed intestine of the rat. To determine whether similar changes happen in obstructed mouse intestine, RT–PCR analysis on RNA extracted from intestinal smooth muscle in control and obstructed mice was performed. As shown in Figure 4A, TNF-α mRNA was expressed in both control and obstructed groups and the ratio of TNF-α mRNA expression to that of GAPDH was significantly increased in obstructed mouse intestine (Fig. 4B).

### Effect of TNF-α on mSCF Expression in Cultured ISMCs

**Figure 5:**
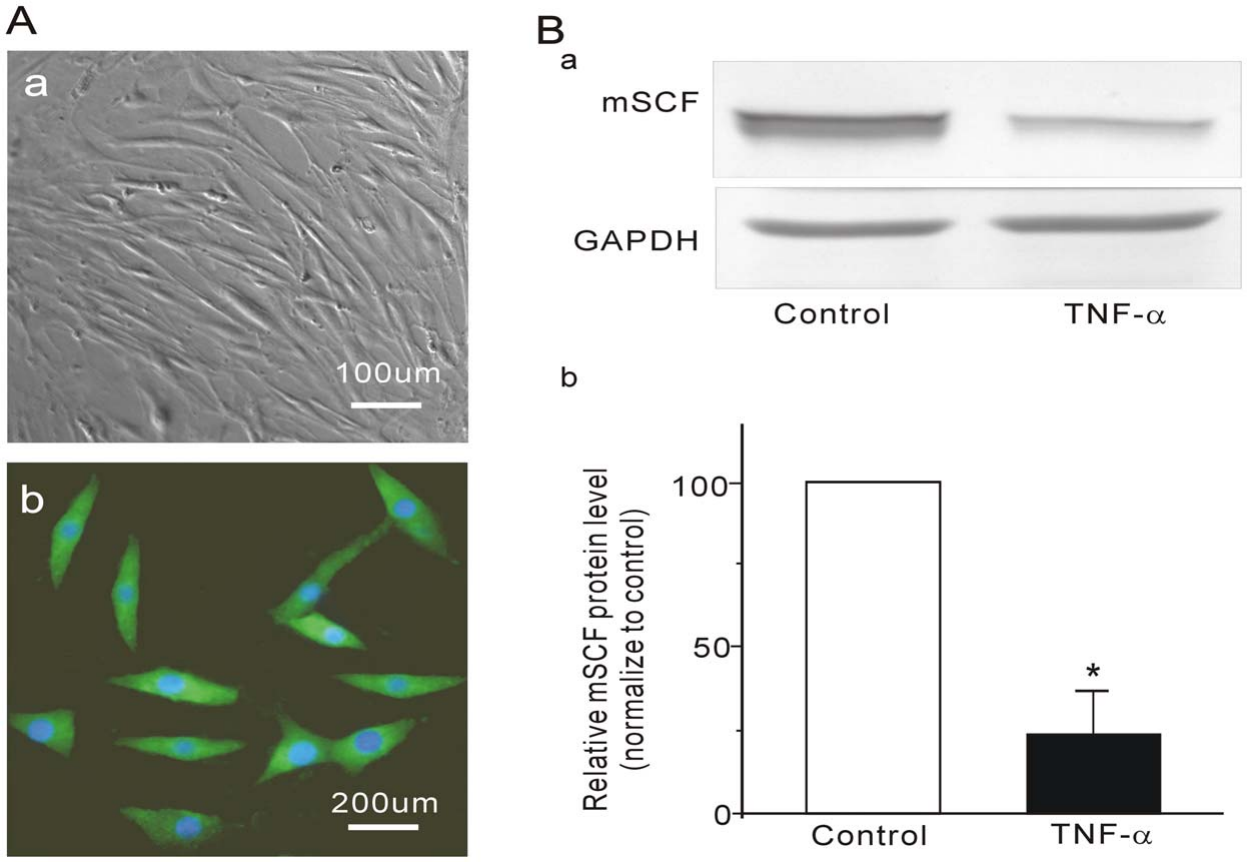
Effect of TNF-α on mSCF expression in cultured intestinal smooth muscle cells. Primary cultures of the intestinal smooth muscle cells are shown in (A). Intestinal smooth muscle cells displayed typical spindle-shaped morphology and a “hill-and-valley” pattern of growth (A-a). Immunofluorescence analysis of cultured intestinal smooth muscle cells revealed smooth muscle α-actin (visualized with FITC-conjugated secondary antibody). DAPI was used to stain the nuclei (A-b). Western blotting revealed that expression of mSCF was significantly decreased in cultured intestinal smooth muscle cells treated with TNF-α (10 ng/ml; B). A typical Western blot result is shown in (B-a). Bands were quantified using densitometry (compared with GAPDH and normalized to control; B-b). **P<0.01, n = 8.

To determine whether partial obstruction-induced decrease in mSCF expression was mediated by up-regulation of TNF-α, ISMCs were treated with TNF-α. Cultured ISMCs displayed typical spindle-shaped morphology and a “hill-and-valley” pattern of growth (Figure 5A-a). ISMCs were also characterized by the expression of smooth muscle-specific α-actin (Fig. 5A-b). Anti-SCF antibody was used to detect mSCF protein in TNF-α and vehicle-treated ISMC preparations. Representative immunoblots for mSCF and GAPDH from TNF-α-treated and vehicle control preparations are shown in Figure 5B-a. Immunoblots for mSCF suggested that mSCF protein existed in the ISMCs. The ratio of corresponding mSCF/GAPDH was calculated and normalized to control. The relative expression of mSCF protein was significantly decreased in TNF-α-treated ISMC preparations compared to that in vehicle control (Fig. 5B-b).

### Effect of Partial Obstruction on CBS and CSE Expression

**Figure 6:**
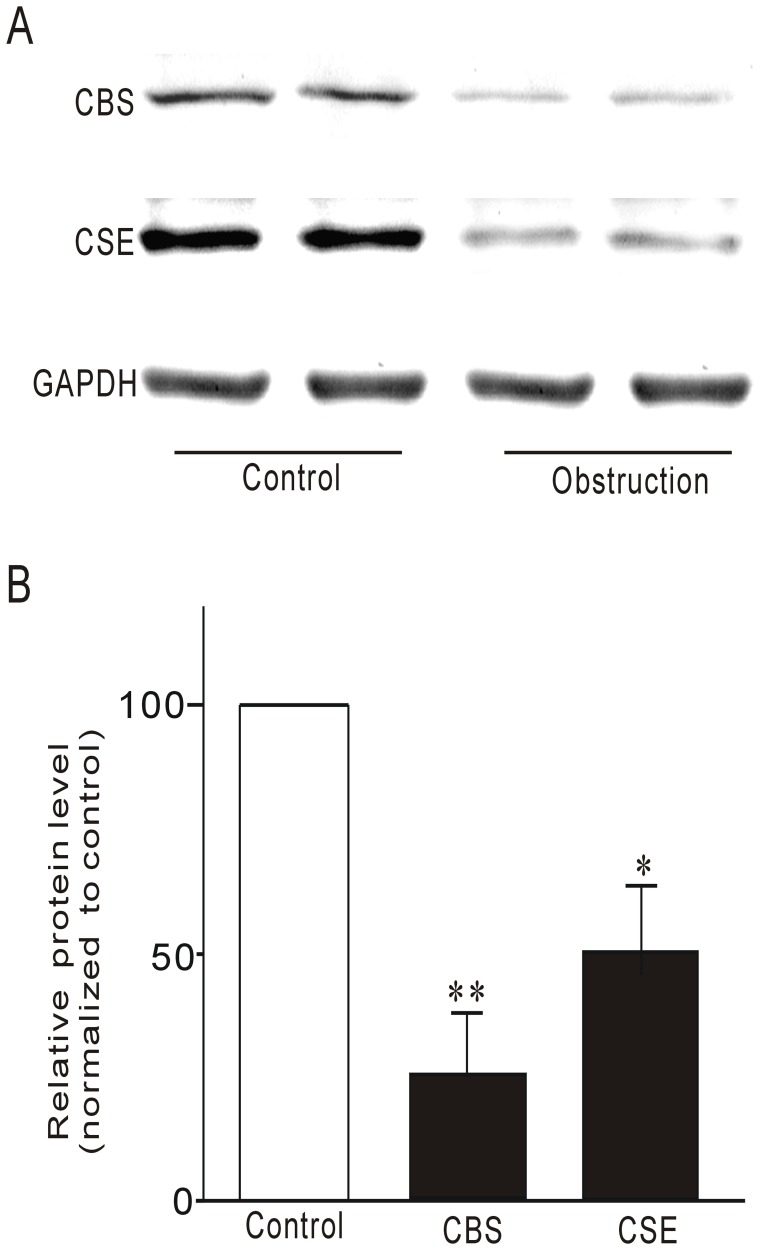
Western blot revealed decreased expression of CSE and CBS in obstructed ileal smooth muscle tissues. A representative blot is shown in (A), whilst the densitometric quantification (%GAPDH and normalized to control) is depicted in (B). **P<0.01, n = 8.

H_2_S has been suggested to be proinflammatory, H_2_S donating compounds have been shown to induce inflammation [24]. In addition, inhibitors of endogenous H_2_S production suppress inflammation [20]. In some situations, H_2_S appears to be anti-inflammatory. The anti-inflammatory effect of H_2_S may be indirect, either due to its tissue-protective effect or hypothermic effect. However, H_2_S may also have direct anti-infllammatory effect by acting directly on the immune system [24], [25]. Since the inflammatory mediator TNF-α was increased in obstructed intestinal smooth muscle tissue, we investigated whether endogenous H_2_S played a role. The expression of enzymes for H_2_S biosynthesis, CSE and CBS, in the obstructed ileum was determined using Western blot. Representative immunoblots for CSE, CBS and GAPDH from control and obstructed groups are shown in Figure 6A. Relative expression of CSE and CBS proteins was significantly decreased in the obstructed group (Fig. 6B).

### Effects of Suppressing H_2_S Biosynthesis on the Expression of TNF-α

**Figure 7:**
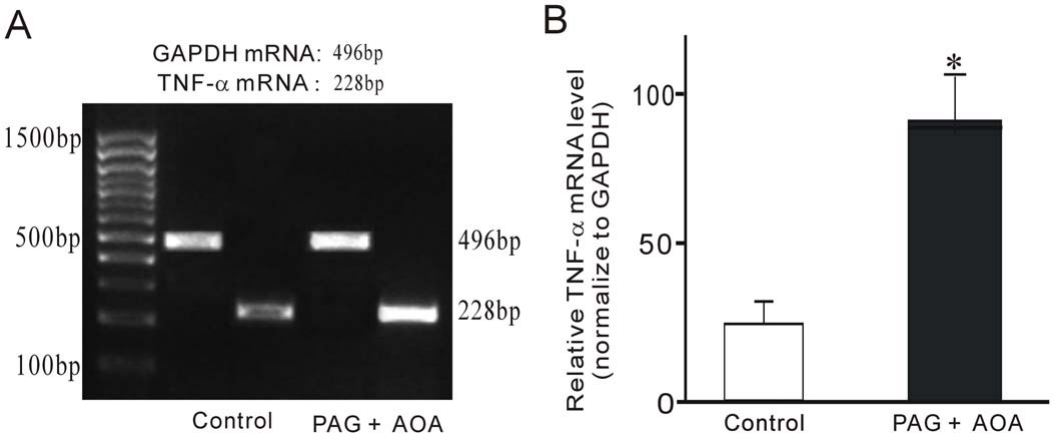
Effect of PAG and AOA on TNF-α mRNA expression in ileal smooth muscle tissues. (A) Expression of TNF-α and GAPDH mRNA. (B) Densitometric analysis of TNF-α and GAPDH mRNA. **P<0.01, n = 8.

To further investigate whether suppression of endogenous H_2_S biosynthesis leads to the increase of TNF-α mRNA in obstructed intestine, the inhibitors of CSE and CBS, PAG and AOA, were administrated via intraperitoneal injection. Our results demonstrated that inhibiting H_2_S biosynthesis by AOA and PAG enhanced the expression of TNF-α mRNA in small intestinal tissue (Fig. 7B), which suggests that endogenous H_2_S may be anti-inflammatory in normal gastrointestinal tract and down-regulation of H_2_S biosynthesis induces increase of TNF-α expression in obstructed intestinal tissue.

### Effect of Supplementation of H_2_S on the Expressions of TNF-α mRNA, mSCF and c-kit Proteins in Obstructed Ileal Smooth Muscle Tissues

**Figure 8:**
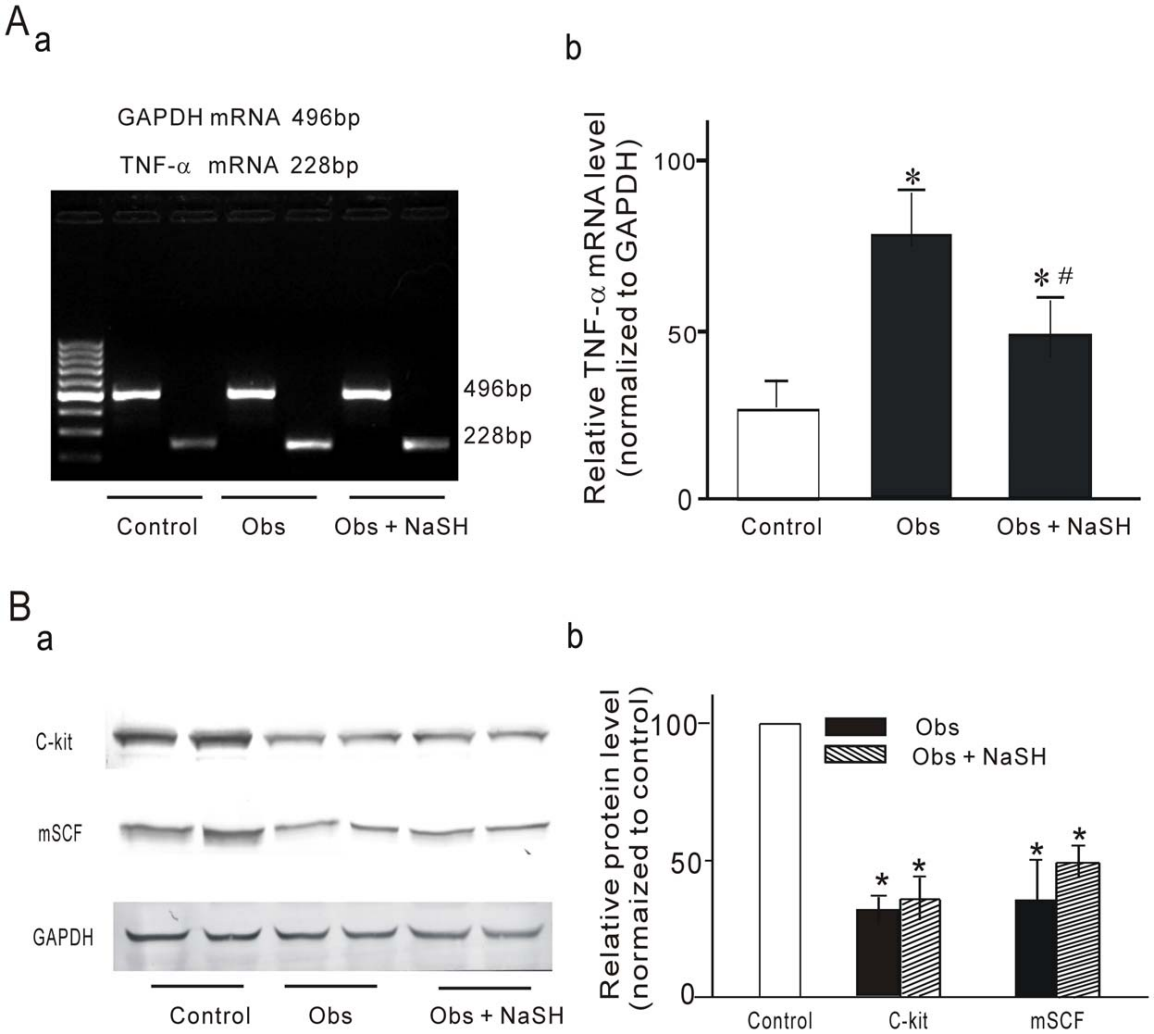
Effect of supplementation of H_2_S on the expressions of TNF-α mRNA, mSCF and c-kit proteins in the obstructed ileal smooth muscle tissues. **A** shows the effect of supplementation of NaHS on TNF-α mRNA expression in obstructed intestinal smooth tissue. (Aa) Expression of TNF-α and GAPDH mRNA. (Ab) Densitometric analysis of TNF-α and GAPDH mRNA. **B** shows the effect of supplementation of NaHS on mSCF and c-kit protein expressions in obstructed intestinal smooth tissue. Representative western blot revealed different expression of mSCF and c-kit (Ba), whilst the densitometric quantification (%GAPDH and normalized to control) is depicted in (Bb). *P<0.01 *vas* control group; # *P*<0.01 *vas* obstructed group. (n = 8)(obs: obstruction).

To further investigate whether supplementation of exogenous H_2_S leads to improve over expression of TNF-α mRNA and enhance expressions of mSCF and c-kit proteins in the obstructed ileal smooth muscle tissues, NaHS were administrated via i.p. The results demonstrated that supplementation of exogenous H_2_S with NaHS significantly improved over expression of TNF-α mRNA (n = 8, *P*<0.01, Fig. 8A). However, the expressions of mSCF and c-kit proteins have an increasing tendency but not significantly restored (n = 8, *P*>0.05, Fig. 8B). The results suggest that endogenous H_2_S may be play an anti-inflammatory effect in normal gastrointestinal tract so down-regulation of H_2_S biosynthesis is related to over expression of TNF-α in obstructed intestinal tissue. In despite of previous study indicated that TNF-α involved in inflammation-induced loss of ICC [8], however, over expression of TNF-α is not one and only reason of loss ICC in obstructed intestinal smooth muscle tissue because obstruction is a complicated pathophysiologic process.

## Discussion

Thickening of the tunica muscularis in the gastrointestinal segment oral to an obstruction is a characteristic adaptation of the digestive tract in response to increased functional demands. The greatest increase in wall thickness occurs close to the obstruction, and results from both smooth muscle cell hyperplasia and hypertrophy. Gabella [4] and Won et al [8] reported that the intestinal walls of guinea pigs and rats with obstructed intestine showed hypertrophy proximal to the region of obstruction. In examinations of mouse models of intestinal obstruction, Chang et al [5] also reported that increase in the thickness of both circular and longitudinal muscle layers of the small intestine was due to increase in the number of cells. In the present study we also observed that obstructed mice intestine exhibited a marked increase in the thickness of the smooth muscle layers in the dilated region compared to those of control mice. These observations confirmed the morphological changes in intestinal smooth muscle layers presented previously in detail by Chang et al [5].

Since obstructed intestinal smooth muscle displayed distinctive structural remodeling, we continued to observe the mechanical and electrical activities of obstructed smooth muscle tissues in vitro. Our results demonstrated that both the frequency and the amplitude of slow waves were significantly decreased. Resting membrane potential of intestinal smooth muscle cells was depolarized 14 days after obstruction. These results were similar to previous studies [5], [8]. These morphological and functional changes also indicated that this animal model is an appropriate study tool for investigations into consequences of intestinal obstruction.

To determine the relationship between ICCs and electrical dysfunction of obstructed smooth muscle, c-Kit expression was investigated. Maintenance of ICCs requires mSCF produced locally within the tunica muscularis [6], [28], [31]. Thus, in the present study, the expression of mSCF and c-Kit were observed in partially obstructed murine intestine. The results showed that both c-Kit and mSCF protein expression was clearly decreased in partially obstructed intestinal smooth muscle layers. ICC depletion may therefore be central to the pathogenesis of motility disorders. Previous studies in a model of obstructed rat ileum also demonstrated that disordered electrical slow waves were accompanied with the disruption of ICCs, along with the disruption of the corresponding network [5]. Consistent with these findings, our results suggest that obstruction-induced electrical dysfunction may be related to the loss of ICCs. Takeda et al [32] observed that reduction in the content of mSCF was also demonstrated in the stomach of non-obese diabetic (NOD) mice, which showed drastic depletion of ICCs and a one-third reduction in the expression of SCF. Reduction in both ICCs and expression level of SCF mRNA was more pronounced in NOD mice than in db/db mice. In the present study, the obstruction-induced reduction in c-Kit expression was accompanied with decreased mSCF expression in obstructed smooth muscle tissue, suggesting that obstruction-induced reduction of c-Kit expression was related to down regulation of mSCF expression in obstructed mice. It is unclear why mSCF expression in smooth muscle was suppressed since intestinal smooth muscle was significantly hypertrophic 14 days after partial obstruction.

mSCF serves as a link between smooth muscles and ICCs. Decrease in mSCF with smooth muscle atrophy underlies ICC loss in diabetic gastropathy and gastroparesis [6]. However, ICC loss in partial mechanical obstruction is known to accompany conditions in which smooth muscle mass is not reduced, but increased [6]. Further investigation into the mechanisms behind the reduction in mSCF protein expression is required. Toshihiko et al [16] reported that inflammation in smooth muscle is central to the loss of ICCs in a Hirschsprung disease model, in which animals have significant intestinal distension (megaileum). This distension was accompanied with the loss of ICCs proximal to a constricted region of the gastrointestinal tract lacking enteric ganglia. TNF-α was reported to have proinflammatory roles in this process and was significantly increased in the obstructed ileum [8]. We hypothesize that TNF-α might be involved in the reduction of mSCF expression in obstructed ileum. To test this hypothesis, we investigated the change of TNF-α in obstructed ileum and the effect of TNF-α on mSCF expression in cultured ISMCs. We found that TNF-α mRNA expression was significantly increased in the distended ileum in the obstructed group. TNF-α treatment suppressed the expression of mSCF protein in cultured ISMCs. These results suggest that obstruction-induced loss of ICCs or reduction of c-Kit expression is due to TNF-α-mediated suppression of mSCF expression.

Although we demonstrated that TNF-α reduced the expression of mSCF, the molecular mechanisms underlying this process need further investigation. H_2_S has been demonstrated as an anti-inflammatory factor [21], [22], [25]. We therefore investigated the expression of H_2_S biosynthesis enzymes CBS and CSE to determine the role of endogenous H_2_S during partial obstruction-induced inflammation. Our experiments demonstrated that CBS and CSE were both down-regulated while TNF-α expression was up-regulated in the same obstructed ileum. To further confirm the relationship between H_2_S and TNF-α, we investigated the expression of TNF-α mRNA in small intestinal tissue after administerations of PAG, an inhibitor of CSE, and AOA, an inhibitor of CBA, via intraperitoneal injection. Inhibition of H_2_S biosynthesis by PAG and AOA up regulated the expression of TNF-α mRNA in intestinal tissue [33]. Our results suggest that down-regulation of H_2_S biosynthesis might be involved in obstruction-induced loss of ICC via up-regulation of TNF-α expression, with the latter suppression of mSCF expression in intestinal smooth muscle. We further investigate the role of supplementation of exogenous H_2_S by i.p NaHS (a H_2_S-releasing agent) in obstruction-induced over expression of TNF-α mRNA and down expressions of mSCF and c-kit proteins. The results demonstrated that supplementation of exogenous H_2_S significantly improved obstruction-induced over expression of TNF-α mRNA, however, did not significantly enhanced the expressions of mSCF and c-kit proteins. Previous study confirmed that TNF-α involved in inflammation-induced loss of ICC [8] and our present study also observed TNF-α can suppress mSCF expression in cultured intestinal smooth muscle cells. Our results suggest that down-regulation of H_2_S biosynthesis might be involved in obstruction-induced loss of ICC via up-regulation of TNF-α expression, with the latter suppression of mSCF expression in intestinal smooth muscle. However, obstruction is very complicated pathophysiologic process so over expression is not one and only reason of loss ICC in obstructed intestinal smooth muscle tissue. Therefore, in despite of supplementation of exogenous H_2_S significantly improved obstruction-induced over expression of TNF-α but not significantly restored the expressions of mSCF and c-kit proteins.

In summary, chronic partial mechanical obstruction of the small intestine induced a smooth muscle remodeling response to obstructive injury, for example, hyperplasia and hypertrophy, and was accompanied by electrical slow wave dysfunction. Obstruction-induced loss of ICCs may be related to mSCF down-regulation mediated by TNF-α; TNF-α up-regluation is possibly induced by down regulation of endogenous H_2_S biosynthesis in obstructed small intestine. However, over expression of TNF-α is not one and only reason of loss ICC in obstructed small intestine, many factors involved in obstruction-induced down expressions of mSCF and c-kit proteins.

## Supporting Information

Figure S1 **Morphological changes and H&E stained in control and obstructed ileum.** The open abdomen and dissection of the entire gastrointestinal tract of a control mouse (A-a). An arrow points to the portion of intestine bearing an opened ring of silicon tubing. 14 days after the creation of a partial obstruction (B-a). An arrow points to the portion of obstructed intestine bearing a ring of silicon tube and surgical thread. Cross-section of the ileum in control (A-b) and obstructed (B-b) mouse tissue stained with hematoxylin-eosin (H&E). In the obstructed intestine, there was a marked increase in the diameter of the ileum (C-a) and a marked increase in the thickness of both the longitudinal and circular muscle layers (C-b). Levels of significance compared to controls are indicated by asterisks (**P<0.01, n = 8).
(TIF)
