# Correction: Down-Regulation of Hydrogen Sulfide Biosynthesis Accompanies Murine Interstitial Cells of Cajal Dysfunction in Partial Ileal Obstruction

**DOI:** 10.1371/annotation/899e2c03-5dfa-41f0-a565-9dd0ddfb512f

**Published:** 2013-05-08

**Authors:** Xin Guo, Xu Huang, Yi-song Wu, Dong-hai Liu, Hong-li Lu, Young-chul Kim, Wen-xie Xu

There was an error in the name of the sixth author.

The correct author name is: Young-chul Kim 

