# Supplementary figures and images for: Down-Regulation of Hydrogen Sulfide Biosynthesis Accompanies Murine Interstitial Cells of Cajal Dysfunction in Partial Ileal Obstruction
Source: PLoS One. 2012 Nov 1;7(11):e48249. doi: 10.1371/journal.pone.0048249 (PMC3486862; doi:10.1371/journal.pone.0048249)

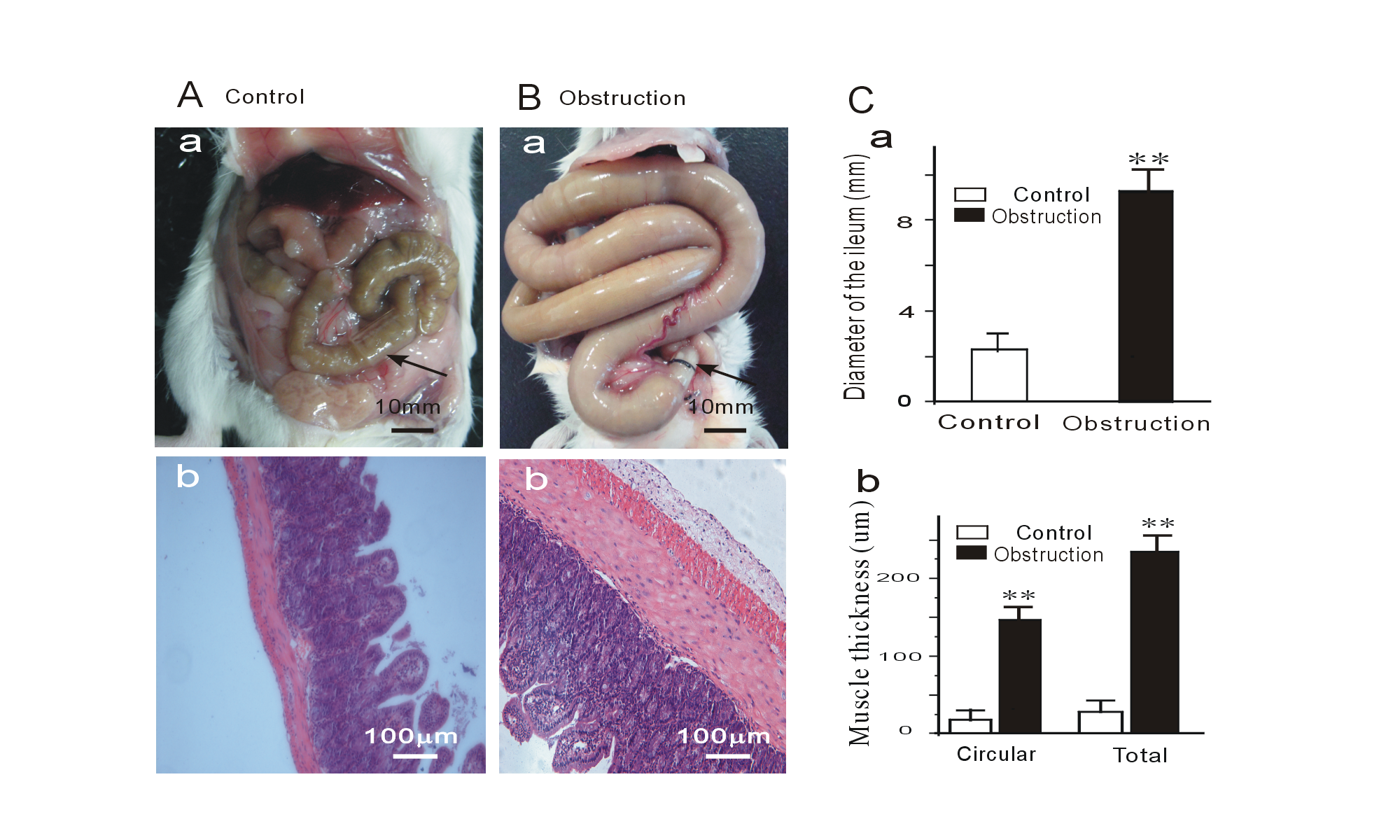

Supplement: Figure S1 — Morphological changes and H&E stained in control and obstructed ileum. The open abdomen and dissection of the entire gastrointestinal tract of a control mouse (A-a). An arrow points to the portion of intestine bearing an opened ring of silicon tubing. 14 days after the creation of a partial obstruction (B-a). An arrow points to the portion of obstructed intestine bearing a ring of silicon tube and surgical thread. Cross-section of the ileum in control (A-b) and obstructed (B-b) mouse tissue stained with hematoxylin-eosin (H&E). In the obstructed intestine, there was a marked increase in the diameter of the ileum (C-a) and a marked increase in the thickness of both the longitudinal and circular muscle layers (C-b). Levels of significance compared to controls are indicated by asterisks (**P<0.01, n = 8). (TIF) [file pone.0048249.s001.tif]
